# Electronic Vaping-Induced Methicillin-Sensitive *Staphylococcus Aureus* Pneumonia and Empyema

**DOI:** 10.1155/2021/6651430

**Published:** 2021-03-06

**Authors:** Sachin M. Patil, Phillip Paul Beck, Tarang Pankaj Patel, Richard Dale Swaney, Dima Dandachi, Armin Krvavac

**Affiliations:** ^1^Department of Medicine, Division of Infectious Disease, University of Missouri Hospital and Clinic, 1 Hospital Dr, Columbia, MO 65212, USA; ^2^Department of Medicine, Division of Pulmonary, Critical Care and Environmental Medicine, University of Missouri Hospital and Clinic, 1 Hospital Dr, Columbia, MO 65212, USA; ^3^Department of Medicine, PGY3 Internal Medicine Resident, University of Missouri Hospital and Clinic, 1 Hospital Dr, Columbia, MO 65212, USA

## Abstract

Pneumonia is a severe acute inflammation of the lower respiratory tract due to infectious pathogens. Pathogens responsible include bacteria, viruses, fungi, and parasites. Pneumonia categorizations include community-acquired pneumonia (CAP), hospital-acquired pneumonia, and ventilator-associated pneumonia. It is the single most common cause of infection-related mortality in the United States. Among the typical bacterial CAP causes, *Staphylococcus aureus* (*S*. *aureus*) is responsible for less than 5% of all cases. Among the *S*. *aureus*, methicillin-susceptible *S*. *aureus* (MSSA) is slightly more common than the methicillin-resistant *S*. *aureus* (MRSA). CAP caused by *S*. *aureus* is associated with worse clinical outcomes compared to *streptococcal pneumoniae*. Although *S*. *aureus* CAP occurs throughout the year, it is less common except during the influenza season when there is a spike. Multiple studies have stratified risk factors for MRSA infection. MSSA pneumonia in immunocompetent young patients is uncommon due to healthy host defense mechanisms. However, certain individual risk factors promote infection, such as intravenous drug abuse. Recent multiple research studies implicate increased virulence of *S*. *aureus* in colonized patients after exposure to electronic cigarette vapor exposure (ECVE), resulting in pneumonia. A PubMed search revealed no MSSA community-acquired bacterial pneumonia due to ECVE. We report a 38-year-old female who developed acute MSSA pneumonia, which was complicated by left empyema due to ECVE from JUUL device with third-party compatible cannabidiol pods. The patient completed treatment successfully with a chest tube placement followed by fibrinolysis and intravenous antibiotics.

## 1. Introduction

*Staphylococcus aureus* (*S*. *aureus*) is a virulent pathogen responsible for a multitude of infections in humans. It colonizes the skin and mucosa. It resides in the nares in 30% of the average population [1]. *S*. *aureus* secretes toxins such as exfoliative toxin that results in staphylococcal scalded skin syndrome and toxic shock syndrome toxin1 responsible for toxic shock syndrome. It also causes food poisoning due to enterotoxins. Its virulence is due to surface adhesins, toxins, enzymes, superantigens, and multiple immune evading mechanisms. The most common clinical infections include skin and soft tissue, bloodstream, and lower respiratory infections. The incidence of lower respiratory tract infections due to *S*. *aureus* has increased over the last decade, and it is now recognized as a common cause of nosocomial pneumonia [1]. The prevalence of *S*. *aureus* community-acquired pneumonia (CAP) increases in the presence of certain risk factors such as recent antibiotic exposure [1]. In the community, it also commonly presents as post-influenza pneumonia in young patients or sporadically with strains carrying the PantonValentine leukocidin toxin. An increased risk of lower respiratory tract infection due to *S*. *aureus* was suspected in patients with electronic cigarette vapor exposure (ECVE) due to inflammation induced by the chemicals contained in the vapor. Recent publications support this hypothesis by demonstrating an increased virulence among colonized *S*. *aureus* strains in a murine pneumonia model [2–5]. However, a review of the medical literature reveals no instance of methicillin-susceptible *S*. *aureus* (MSSA) pneumonia due to ECVE. Here, we describe a case with acute MSSA pneumonia complicated by left empyema in a patient with significant ECVE from the use of a JUUL device with third-party compatible cannabidiol (CBD) pods.

## 2. Case Presentation

**Figure 1:**
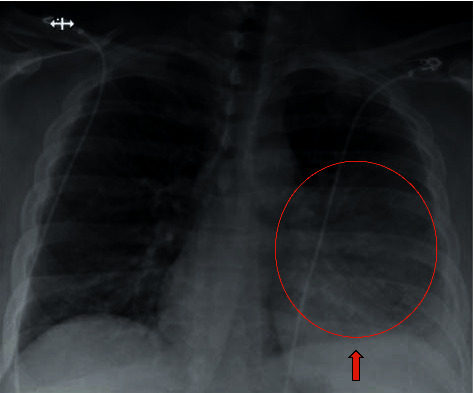
Chest X-ray 2 views revealing left lower lobe pneumonia with trace left pleural effusion with no pneumothorax or osseous abnormalities.

**Figure 2:**
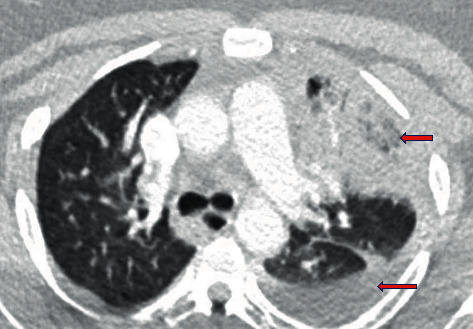
CT chest with contrast revealing extensive left upper lobe and lingular pneumonia with small lef-sided partially loculated pleural effusion.

A 38-year-old female presented to our emergency department with productive cough, fever, night sweats, and generalized weakness for five days. The phlegm was moderate in quantity with a pinkish tinge, nonfoul smelling, and without hemoptysis. The productive cough was accompanied by progressive dyspnea and chest pain. Over the counter, acetaminophen did provide some relief to her fever and chest pain. She denied any recent travel history, illness, sick contacts, hospitalization, vomiting, aspiration, or antibiotic exposure in the last three months. She denied headaches, visual disturbances, and extremity weakness. Her past medical history was significant for asthma as a kid and obesity, with a body mass index of 40 kg/m^2^. She denied illicit substance and alcohol abuse but reported a six-year history of tobacco dependence, which she had quit six months back. Her vaccination history included a 23-valent pneumococcal vaccine. Physical examination revealed a stable blood pressure with a tachycardia of 133 beats per minute and tachypnea of 26 breaths per minute. She was afebrile and saturated 94% on room air. Lung auscultation revealed diminished left lung air entry with crackles over the left upper and lower lobe and increased respiratory effort. There was no presence of skin rash, pharyngeal erythema, or tonsillar enlargement. Complete blood count with differential revealed leukocytosis of 12,830/mL with neutrophils of 83.3% and lymphocytes of 7.6%, whereas a complete metabolic panel was normal. Urine analysis, nasopharyngeal respiratory pathogen panel, and nasopharyngeal influenza A and B antigens were negative. Urine legionella and streptococcal antigen were negative with pending blood cultures. Human immunodeficiency virus and hepatitis serology were negative, and Hemoglobin A1c was 5.5. D-dimer was 1.01 mcg/mL with a normal lipid panel. A chest radiograph showed left lower lobe opacification with a trace left pleural effusion (Figure 1). She received a single intravenous (IV) dose of ceftriaxone and azithromycin. Subsequent computed tomography (CT) scan of the chest revealed significant left lung consolidation with a partially loculated left-sided pleural effusion. It was negative for acute pulmonary embolism (Figure 2). Later in the day, the patient developed a fever of 38.4°C.

**Figure 3:**
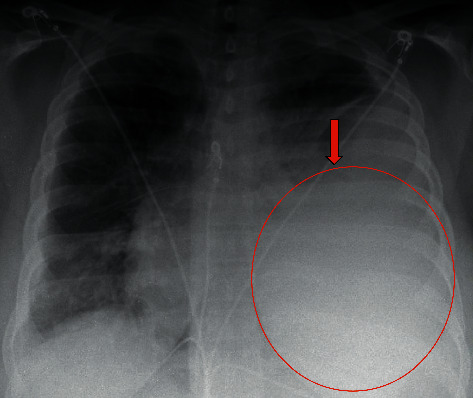
Chest x-ray portable revealing worsening left pneumonia with empyema.

One out of two bottles returned positive for Gram-positive cocci on day two of admission, resulting in an escalation of antibiotics to vancomycin and levofloxacin. The infectious disease (ID) team was consulted because of bacteremia and pneumonia with left parapneumonic effusion. The ID team recommended repeat blood cultures, sputum culture, and left pleural tap for pleural fluid analysis and culture. Blood cultures done at admission returned positive for *coagulase-negative Staphylococcus* species, which was considered a contaminant. Antibiotics were switched back to ceftriaxone and azithromycin. On day three, the patient developed a new oxygen supplementation of two liters via nasal cannula. She continued to have left-sided chest pain, subjective low-grade fever, and night sweats. Serum procalcitonin was high at 11.3 ng/mL, and a repeat chest X-ray disclosed a worsening left opacification (Figure 3). A diagnostic thoracentesis yielded 500 mL of purulent fluid consistent with empyema. The pleural fluid analysis confirmed this, prompting a left chest tube placement and intrapleural fibrinolytic therapy with tissue plasminogen activator and dornase alpha (Tables 1 and 2). Left pleural fluid cytology revealed marked acute inflammation consistent with empyema. On day four, the sputum culture returned positive for Gram-positive cocci, and the patient was started on vancomycin. On day five, oxygen requirements increased to four liters with a repeat chest X-ray revealing worsening empyema. The sputum and pleural fluid culture were positive for MSSA on day six, prompting subsequent antibiotic therapy de-escalation with nafcillin. Thoracic surgery evaluated the patient for video-assisted thoracoscopic decortication (VATS), but ultimately this was not required as the patient had a favorable response to intrapleural fibrinolytic therapy.

**Figure 4:**
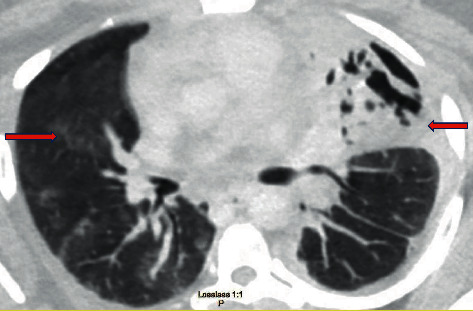
CT chest revealing decrease in left size empyema, interval development of a large focus of necrotizing pneumonia within the right middle lobe, and interval improvement in right lung multifocal pneumonia.

**Figure 5:**
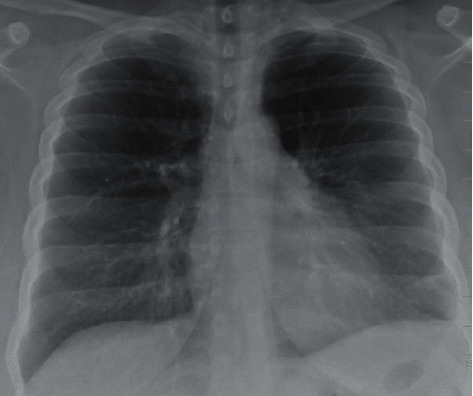
Chest X-ray revealing improved aeration of the lingula and left lower lobe with residual scarring and left pleural thickening.

A repeat CT scan of the chest on day twelve revealed a decrease in the left empyema size with improved aeration of the left and right lung (Figure 4). The chest tube was subsequently removed, and the patient was discharged on a six-week course of IV antibiotics. Further clinical history revealed that the patient had started using an electronic cigarette (JUUL device with compatible third-party CBD pods) for the last six months after quitting smoking. The patient reported vaping about six times daily. She was counseled to discontinue the use of any cigarettes and vaping products. She reported successfully stopping the use of vaping and tobacco products at her follow-up appointment. Her symptoms had resolved, and a repeat chest X-ray disclosed improved aeration of the lingula and left lower lobe with residual scarring and left pleural thickening/fluid (Figure 5).

## 3. Discussion

*S*. *aureus* is a virulent Gram-positive bacterium responsible for causing life-threatening infections such as catheter-related bloodstream infections, surgical site infections, and ventilator-associated pneumonia. In a study on 2259 patients hospitalized for CAP, *S*. *aureus* was responsible for 1.6% of patients [6]. MSSA was responsible for 1% of the cases, and in contrast, MRSA accounted for 0.7% of the cases [6]. MSSA prevalence was 3.9%, while MRSA prevalence was 2.4%. MRSA prevalence is 2.7% in the ICU setting, but on the regular medical floor, it is 2% [6]. An increased prevalence in the ICU justifies the use of empirical MRSA treatment in critically ill CAP patients. Inpatient mortality for MRSA CAP (13.3%) is slightly higher than that for MSSA CAP (9.1%), compared with lower mortality of Streptococcal pneumoniae at 4.4% [6]. Risk factors responsible for an increased risk of MRSA pneumonia are as follows in Table 3 [1, 7]. The risk of *S*. *aureus* invasive infection is higher in the following medical conditions mentioned in Table 4. Persistent nasal carriers are associated with an increased rate of infection, and the elimination of the carrier state has decreased procedure-related nosocomial infections [1]. The incidence of *S*. *aureus* nosocomial pneumonia has gradually increased over the last decade. It now accounts for 20% to 30% of hospital-acquired pneumonia [1]. It is still an unsettled question of whether MRSA is more virulent than MSSA or vice versa.

Compared with other bacterial causes, the clinical features of *S*. *aureus* pneumonia are nonspecific. The transmission methods involved are aspiration or bacteremia-related seeding or airborne mode. *S*. *aureus* pneumonia is a necrotizing infection with brisk pulmonary tissue disintegration followed by cavitation and complications, including abscesses and empyema. In healthy adults and younger patients, it follows an influenza infection characterized by worsening respiratory symptoms after a slight improvement in or of viral pneumonia. Intravenous drug abuse-related MSSA pneumonia is due to bacteremia or infective endocarditis. Younger males and elderly individuals are at higher risk for MSSA pneumonia. *S*. *aureus* pneumonia clinical outcomes are worse with a prolonged hospital stay and an increased likelihood of admission to an intensive care unit [6]. The case fatality rates of community-acquired MRSA infection can go up to 60% [1].

Electronic cigarette (e-cigarette) use among youth has become widespread due to the perception that it is safer than cigarette smoking. In 2019, approximately more than five million students were actively using e-cigarettes, including 10.5% of middle school and 27.5% of high school students [8]. In adults, e-cigarettes have been used as a smoking cessation aid. Most e-cigarette users above 45 are prior or current smokers, whereas, in the younger group, very few have a previous smoking history. The e-cigarettes have a pod that contains nicotine or flavored nicotine from the manufacturer. However, lately, multiple third-party manufactures have made pods containing CBD oil, hemp, or kava, which are compatible with numerous vaping devices. These third-party pods are not monitored by any agency to check the safety of the components. Furthermore, noteworthy is that these pods' nicotine quantity is variable and frequently higher than that observed in combustible cigarettes. Unlike cigarette smoking, it is difficult to quantify how much nicotine a person is vaping via an e-cigarette. Due to the availability of CBD and other addictive components in these pods, their use is more frequent now for recreational purposes. As these third-party pods are cheap, contain more e-liquid, and offer more nicotine and flavor options, their popularity has skyrocketed.

E-cigarette device heats the e-liquid and delivers aerosolized vapor to the lungs. E-liquids contain three main ingredients: the vehicle mixture, flavoring agents, and nicotine in the form of salt. The vehicle mixture of humectant contains propylene glycol or vegetable glycerin. Flavoring agents used are cinnamaldehyde, diacetyl, 2, 3-pentanedione, acetoin, and maltol. Nicotine concentration varies from 0–36 mg/ml [9]. The aerosolized vapor contains numerous respiratory irritants and toxicants, such as volatile organic compounds, acrolein, and formaldehyde. The chemicals in electronic cigarette vapor are cytotoxic, increase mucin production, and induce proinflammatory cytokines and proteases. This effect culminates in increased airway hyperreactivity and suppressed mucociliary clearance [9]. The result is the impairment of antimicrobial defenses and the destruction of lung tissue. One example of this result is the e-cigarette or vaping use-associated lung injury (EVALI). EVALI imaging has revealed different radiographic presentations [10]. EVALI has reached epidemic proportions in the United States with close to 2,800 patients until February 2020 as per the Center for Disease Control and Prevention. One of the agents suspected was vitamin *E* acetate used as an additive thickening agent in some illicit CBD vape cartridges.

There is no difference in nicotine absorption between traditional and electronic cigarettes. Nicotine is a toxic chemical that enhances *S*. *aureus* initial attachment to epithelial cells and biofilm formation [11]. It transiently suppressed *S*. *aureus* virulence and diminished its ability to invade epithelial cells. Biofilms result in *S*. *aureus* persistence in the airway by preventing host attacks. Nicotine's net effect is an increased *S*. *aureus* fitness and its adaptation to the upper airway, contributing to chronic infection [11]. Epidemiological studies reveal a higher risk of invasive *S*. *aureus* infections, including MRSA pneumonia in chronic smokers. The cigarette smoke exposure (CSE) effect is directly related to the degree of exposure and the colonizing *S*. *aureus* strain genetic background [2]. CSE promotes biofilm formation with reduced toxin expression and persistence of *S*. *aureus*. Cellular stress induced by CSE on both the host and the microorganism results in a mutagenic effect with the emergence of small colony variants (SCVs) and antibiotic resistance. Once acclimatized to host, SCVs by their enhanced invasive behavior and persistence are responsible for recurrent infections.

ECVE effect on the respiratory airway microbiome is unknown [2]. Similar to CSE, ECVE has significant airway effects resulting in diffuse cellular damage and disrupted lung innate defense mechanisms. On airway epithelial cells, it is cytotoxic and reduces the keratinocyte antimicrobial activity. It also breaks the endothelial barrier and incites inflammation. ECVE diminished the macrophage antimicrobial activity, but the neutrophil antimicrobial activity decrease was in a nicotine concentration-dependent manner. On MRSA, ECVE had a myriad of effects. They turn hydrophobic with an increase in the ability to adhere, invade, and persist within the keratinocytes [3]. An increase in virulent gene expression and resistance to the human cathelicidin antimicrobial peptide LL-37 is observed in MRSA [3, 4]. In the murine MRSA pneumonia model, mice were exposed to ECVE for 60 minutes once daily for four weeks. ECVE resulted in significant changes in bronchoalveolar lavage (BAL) of these mice. ECVE resulted in an elevation of three BAL proinflammatory cytokines, keratinocyte chemoattractants, interleukin receptor 1 antagonist, and triggering receptor expressed on myeloid cells 1 by 10% [3]. BAL also disclosed a decrease of >50% of two cytokines, interleukin-3 (IL-3), and granulocyte-macrophage colony-stimulating factor (GM-CSF) [3]. Pentraxin 3, an acute phase reactant in serum, was elevated with no change in the BAL cellularity and differential [3]. Pentaxin 3 elevation is seen in systemic inflammatory conditions, such as stroke and coronary heart disease. A decline in BAL IL-3 and GM-CSF increases vulnerability to fungal and bacterial infections [3]. In infected mice, MRSA upregulated its virulence factors coa (code for coagulase) and PVL (code for Panton-Valentine leukocidin) [3].

In a study conducted to evaluate the effect of ECVE on common respiratory pathogens, biofilm formation in *S*. *aureus* was higher with ECVE compared to CSE [4]. Interleukin-8 and tumor necrosis factor-alpha levels were higher in *S*. *aureus* ECVE compared to CSE, indicative of higher inflammation [5]. The mechanisms involved in inflammation post-ECVE match the one seen with bacterial infection or bacteria exposed to CSE. This data indicate that *S*. *aureus* infection in vapers might be lethal with difficulty in treatment. In another study, *Streptococcus pneumoniae*, ECVE, or CSE resulted in a modest biofilm formation with no hydrophobicity changes or epithelial cell adherence [9]. There is a significant disparity in the puff profile between vapers and conventional cigarette users. Vaping pods contain added nicotine in comparison, and vapers take long hefty puffs increasing nicotine delivery to the airway. In addition, flavoring agents and additives used in e-liquid might have harmful effects on the airway and innate lung defense mechanisms. Thus, ECVE declines a host's inherent resistance to infection while promoting airway bacterial colonization and virulence, resulting in inflammatory lung disease.

Our patient had no individual risk factors for *S*. *aureus* pneumonia and empyema except for the patients' vaping behavior. The delirious effects of vaping added to *S*. *aureus* increased the virulence on exposure to ECVE resulted in her having a left empyema with multifocal necrotizing pneumonia. A PubMed literature review revealed no prior cases of MSSA necrotizing pneumonia after ECVE. Our case is the first in the medical literature. As explained above, ECVE induced pulmonary injury and subsequent BAL changes predispose an individual to bacterial and fungal infections. It is necessary to ask questions with regard to vaping in younger patients with pneumonia. It raises a question, should we cover for MSSA and MRSA in a young vaper with pneumonia admitted to the medical floor. The current guidelines recommend MSSA or MRSA coverage in a community-acquired bacterial pneumonia patient admitted to the intensive care unit only [12]. We suggest considering MSSA and MRSA coverage in a young patient with a vaping history if the clinical status does not improve in 48 hrs, and imaging studies indicate worsening pneumonia with complications. Our patient quit vaping and completed successful treatment with iv antibiotics, chest tube, and fibrinolytic. A recent study reveals an increasing change in attitudes about e-cigarettes and vaping product safety, which can be attributed to public education and media attention about EVALI [13].

## 4. Conclusion

*S*. *aureus* pneumonia in young adults is uncommon except in viral infections and patients with risk factors for invasive disease. It is crucial to detect the predisposition given the case fatality associated with necrotizing pneumonia in patients with no identifiable risk factors. This case unravels the importance of obtaining an excellent clinical history at admission regarding the patient's history of smoking and recreational substance abuse. It has become prudent to inquire about vaping exposure, particularly among younger patients with pneumonia. This case also highlights the fact that vaping causes structural lung injury and predisposes to bacterial infections. We believe that *S*. *aureus* pneumonia among e-cigarette users is underreported and encourage more reporting of pneumonia from ECVE.

## Figures and Tables

**Table 1:** Pleural fluid analysis.

Appearance and color	Brown and cloudy
Glucose	<2 mg/dL
Total protein	4.7 gm/dL
Lactate dehydrogenase (LDH)	1,478 units/L
White blood cells	5,082/mcL
Total nucleated cells	5,083/mcL
Red blood cell	<3000/mcL
Neutrophils	100%

**Table 2:** Lights criteria: exudative effusions will have at least one or more of the following.

	Patient
Pleural fluid protein/serum protein (6.7 gm/dL) > 0.5	0.7
Pleural fluid LDH/serum LDH (285 units/L) > 0.6	5.18
Pleural fluid LDH > 2/3 serum LDH upper limit of normal	Yes

**Table 3:** Risk Factors for invasive *S*. *aureus* infection.

(i) Hemodialysis
(ii) Peritoneal dialysis
(iii) Human immunodeficiency virus infection
(iv) Solid-organ transplantation
(v) Heart disease
(vi) Cancer
(vii) Illicit intravenous drug use
(viii) Alcohol abuse
(ix) Diabetes mellitus
(x) Stroke
(xi) Chronic obstructive pulmonary disease (COPD)
(xii) Systemic lupus erythematosus
(xiii) Rheumatoid arthritis

**Table 4:** Risk factors for MRSA pneumonia.

(i) Recent hospitalization
(ii) Recent antibiotics
(iii) Tobacco use
(iv) Illicit drug use
(v) COPD
(vi) Liver disease
(vii) Human immunodeficiency virus infection

## Data Availability

The data used to support this study are available within the article.
